# Psychological Effects on Health Science Students After Implementation of COVID-19 Quarantine and Distance Learning in Saudi Arabia

**DOI:** 10.7759/cureus.11767

**Published:** 2020-11-29

**Authors:** Sultan Qanash, Faisal Al-Husayni, Shereen Alemam, Lina Alqublan, Emad Alwafi, Hani N Mufti, Husam Qanash, Mohammed Shabrawishi, Ala’a Ghabashi

**Affiliations:** 1 Internal Medicine, National Guard Hospital, King Abdulaziz Medical City, Jeddah, SAU; 2 Medicine, King Abdullah International Medical Research Center, Jeddah, SAU; 3 Medicine, King Saud Bin Abdulaziz University for Health Sciences, Jeddah, SAU; 4 Internal Medicine, King Abdullah International Medical Research Center, Jeddah, SAU; 5 Respiratory Therapy, King Abdullah Medical City Specialist Hospital, Makkah, SAU; 6 Radiology, King Fahad Armed Forces Hospital, Jeddah, SAU; 7 Cardiac Surgery, King Faisal Cardiac Center, King Abdullah Medical City, Jeddah, SAU; 8 Medicine, University of Hail, Hail, SAU; 9 Internal Medicine, Alnoor Specialist Hospital, Makkah, SAU; 10 Intensive Care, National Guard Hospital, King Abdulaziz Medical City, Jeddah, SAU

**Keywords:** psycho-behavioral, health sciences, covid 19, coronavirus quarantine, distance learning programs, anxiety, depression, health professions students, saudi arabia

## Abstract

Background

The 2019 novel coronavirus disease (COVID-19) pandemic has impacted the globe dramatically. It has affected daily life noticeably and the teaching process is one of the significantly affected aspects as the learning approach has been shifted to distance learning (DL). These new changes may affect student performance and emotional well-being. This study aimed to assess the psychological impact of the COVID-19 pandemic and distance learning on healthcare students.

Method

An online self-administered cross-sectional survey was distributed to healthcare students for the period between April 2020 to June 2020. The study included students from different universities in Saudi Arabia. Knowledge and perception of COVID-19 and the experience of distance learning during the pandemic period were assessed using a 5-point Likert scale. Psychological effect was evaluated using Four-Item Patient Health Questionnaire for Anxiety and Depression (PHQ-4).

Results

A total of 721 students completed the survey with the majority being females. Around 25% of students had experienced anxiety, while 35% had depression. Severe anxiety and depression were noted in over 6% of the participants. Younger age and female gender were more affected psychologically. Students with higher scores in PHQ-4 were strongly disagreeing that hand gloves and surgical masks may help in preventing COVID-19 transmission. Students with normal psychological assessment were more likely to favor DL, while students with moderate to severe anxiety and depression disagreed.

Conclusion

Healthcare students have alarmingly high levels of anxiety and depression during the COVID-19 pandemic. General knowledge of the pandemic is not associated with the psychological impact. DL is a convenient approach for students with normal PHQ-4 scores. Programs to help students overcome the psychological impact of COVID-19 are highly recommended.

## Introduction

New learning approaches are continuously required in medical teaching settings to enhance scholastic and organizational improvement. Community-based medical education is a well-known method that has been expanding and taking over the traditional academic medical system [1]. An example of community-based education is distance learning (DL), which is defined as any learning process where the teacher or tutor is geographically apart from the students; or when students are separated from each other or learning resources [2,3]. As the technology is evolving, delivering scientific knowledge and medical expertise to students is becoming easier and timely achieved [4]. Compared to traditional methods, DL provides many advantages such as 24-hour access to the materials, opportunity for students to learn at a desirable pace and increasing the students’ capacity in educational institutions [3]. These advantages deliver a generous learner-centered educational development.

Since the beginning of the coronavirus disease 2019 (COVID-19) pandemic, Saudi Arabia has witnessed massive transformations to tackle the disease spread. These regulatory changes were implemented and affected all education disciplines in the country. In February 2020, the Saudi government has issued multiple laws including impending curfew, which subsequently lead to closing universities including medical schools [5]. To maintain the learning wheel running during COVID-19 circumstances, many universities diverted the teaching method to DL via online lectures, video conferences, and online exams. Fortunately, DL is not ambiguous to medical education, as it has proven to be efficient [6,7].

The influence of the COVID-19 pandemic is not exclusive to medical education, but it has been linked to many psychological effects among the public. In China, more than 15% of the population suffered from moderate to severe depressive symptoms, and almost one-third experienced symptoms of anxiety. Furthermore, students carried higher rates of anxiety and depression during COVID-19 crises, and it is mostly related to the fear of academic delay [8]. The mental and psychological well-being of college students is significantly affected by public health emergencies, which require social and academic support.

This study aims to evaluate the psychological effect of the COVID-19 pandemic on health science students in Saudi Arabia and also to assess their general knowledge of COVID-19. Moreover, we intend to identify students’ perception of the learning process since the curfew mandated DL in all universities in Saudi Arabia.

## Materials and methods

Participants

This is a cross-sectional survey conducted using self-administered questionnaires for the period between April 2020 to June 2020. The criteria for inclusion were willingness to participate in the study and being a current health sciences student in the western region of Saudi Arabia. Exclusion criteria were failure to complete the questionnaire and a personal history of psychological illness. An informed consent was required before filling the survey. This study gained Institutional Review Board approval from King Abdullah International Medical Research Center, approval #RJ20/064/J.

Questionnaire

The survey is a self-designed questionnaire in which fact validity, content validity, and a pilot study were performed. It consists of four sections. The first part included socio-demographic information. The second and third parts assessed students’ knowledge and perception of COVID-19 and the experience of DL during the pandemic period. Knowledge questions were focusing on disease transmission and infection control measures, while DL perception was concentrating on accessibility and convenience of the teaching method. The questions mainly consisted of 5-point Likert scale. The final section assessed COVID-19 pandemic psychological effect using a validated screening tool, the Four-Item Patient Health Questionnaire for Anxiety and Depression (PHQ-4). It consists of four items; the first two questions are about anxiety and the second two questions are about depression. The total score is the summation of all four questions. Scores are rated as normal (0-2), mild (3-5), moderate (6-8), and severe (9-12). A total score ≥3 for the first two questions and the last two questions suggest anxiety and depression, respectively [9]. 

Data collection

As direct interviews were unobtainable due to curfew regulations, we used an online tool (Google Forms) to distribute the questionnaire. The method sampling was a non-probability convenient sampling technique. The link was sent to all health science students in universities in the western region of Saudi Arabia. The recruitment was completely voluntary.

Statistical analysis

All statistical analyses were performed using R software, version 3.6.2 (R Core Team (2019). R: A language and environment for statistical computing. R Foundation for Statistical Computing, Vienna, Austria. https://www.R-project.org/.). Exploratory data analysis on demographics, knowledge about COVID-19 and perception of distance learning survey questions was done. The PHQ4 anxiety, depression and total score were calculated. Some of the survey questions were on a binary scale (like Gender= Male/Female) and were treated as categorical variables. Other variables were on a Likert type (five levels like Strongly agree, Neutral, Disagree and Strongly Disagree) were treated as ordinal scale.

The mean and standard deviation were used for continuous variables that had a normal distribution and were compared using the Two Sample t-test or Welch Two Sample t-test if the two groups had unequal variance. Continuous variables that were not normally distributed were reported using the median and interquartile range and were compared using the Wilcoxon rank sum test. Categorical variables were reported as frequencies and percentages and were analysed by chi-square or Fisher’s exact test as appropriate. The Kruskal-Wallis test was used for ordinal attributes. For Likert type data, frequencies were used to describe the responses and the chi-square to identify relationships with the total PHQ4 score. All statistical tests were two-tailed and p-values < 0.05 were considered significant.

## Results

General characteristics

A total of 721 students completed the survey. Of these, 59.4% (n=428) were females. The mean age was 22 years (SD = ±2). Around 50% of the participants (n= 362) were from the College of Medicine and just over two-thirds were from senior years (4th to 6th year in school) (Table 1).

**Table 1:** General characteristics of the participants

	n= 721 patients
Age (years)	
Mean (SD)	22 (2)
Female gender, n (%)	428 (59.4)
College, n (%)	
Applied Sciences	186 (25.8)
Medicine	362 (50.2)
Nursing	57 (7.9)
Pharmacy	56 (7.8)
Others	60 (8.3)
School year, n (%)	
1	9 (1.3)
2	64 (8.9)
3	160 (22.2)
4	211 (29.3)
5	134 (18.6)
6	143 (19.8)

Psychological assessment

In Table 2, the PHQ-4 was used to assess the impact of quarantine due to COVID-19 and the abrupt implementation of distance virtual learning on student’s anxiety and depression. Approximately 25% of students had an anxiety score ≥3, which implies the presence of a level of anxiety. However, more students were suffering from depression with around 35% scoring ≥3 in the depression questions of the PHQ-4. For the PHQ-4 total score, just over one-third of students (36.8%) had normal scores of less than 3 and only 6.7% had a score above 8, which suggests the presence of severe anxiety and/or depression.

**Table 2:** Psychological assessment of the participants.

Question	n= 721 patients
Over the LAST 2 WEEKS, how often have you been feeling nervous, anxious or on edge, n (%)	
Not at All	236 (32.7)
Several Days	332 (46.1)
More than half the days	93 (12.9)
Nearly Every day	60 (8.3)
Over the LAST 2 WEEKS, how often have you been unable to stop or control worrying, n (%)	
Not at All	349 (48.4)
Several Days	245 (34)
More than half the days	76 (10.5)
Nearly Every day	51 (7.1)
Over the LAST 2 WEEKS, how often have you been feeling down, depressed or hopeless, n (%)	
Not at All	265 (36.8)
Several Days	270 (37.5)
More than half the days	126 (17.5)
Nearly Every day	60 (8.3)
Over the LAST 2 WEEKS, how often have you been experiencing little interest or pleasure in doing things, n (%)	
Not at All	212 (29.4)
Several Days	308 (42.7)
More than half the days	126 (17.5)
Nearly Every day	75 (10.4)
Anxiety score ≥3, n (%)	178 (24.7)
Depression score ≥3, n (%)	249 (34.5)
PHQ4 Score, n (%)	
Normal (0-2)	265 (36.8)
Mild (3-5)	286 (39.7)
Moderate (6-8)	122 (16.9)
Severe (9-12)	48 (6.7)

General characteristics by PHQ-4 categories

Because of the small number of students in the PHQ-4 moderate and severe categories, these two were combined into one category (Moderate to Severe). Table 3 shows a univariate analysis of demographic variables with the PHQ-4 scores. Younger age and female gender were found to be statistically significant and highly associated with higher scores (P-value <0.001). 

**Table 3:** General Characteristics by Patient Health Questionnaire for Anxiety and Depression (PHQ-4) Categories. * indicates statistical significance <0.05

		PHQ4 Categories				
Participants characteristic	All Patients (n= 721)	Normal (n= 265, 36.8%)	Mild (n= 286, 39.8%)	Moderate to Severe (n= 170, 23.6%)	p-value	
Age (years)						
mean (SD)	22 (2)	22.6 (2.5)	22 (1.9)	22.1 (1.5)	<0.001 *	
Female gender, n (%)	428 (59.4)	121 (45.7)	189 (66.1)	118 (69.4)	<0.001 *	
College, n (%)					0.32	
Applied Sciences	186 (25.8)	56 (21.1)	81 (28.3)	49 (28.8)		
Medicine	362 (50.2)	146 (55.1)	139 (48.6)	77 (45.3)		
Nursing	57 (7.9)	21 (7.9)	23 (8)	13 (7.6)		
Pharmacy	56 (7.8)	16 (6	24 (8.4)	16 (9.4)		
Others	60 (8.3)	26 (9.8)	19 (6.6)	15 (8.8)		
School year, n (%)					0.134	
1	9 (1.3)	7 (2.6)	1 (0.3)	1 (0.6)		
2	64 (8.9)	23 (8.7)	28 (9.8)	13 (7.6)		
3	160 (22.2)	47 (17.7)	70 (24.5)	43 (25.3)		
4	211 (29.3)	72 (27.2)	90 (31.5)	49 (28.8)		
5	134 (18.6)	48 (18.1)	52 (18.2)	34 (20)		
6	143 (19.8)	68 (25.7)	45 (15.7)	30 (17)

Sources of information and perception on distance learning by PHQ-4 category

Analysis of sources of information revealed that students with high scores in PHQ-4 are less likely to use non-formal media (like WhatsApp or Twitter) to seek information and more likely to be not interested in seeking information about COVID-19 when compared to students with normal or mild scores (P-value 0.005 and 0.003, respectively). Around 40% of students had experienced distance learning in the past prior to its implementation in the school (Table 4).

**Table 4:** COVID-19 sources of information and student perception on distance learning by Patient Health Questionnaire for Anxiety and Depression (PHQ-4) Categories. * indicates statistical significance <0.05. MOH: Ministry of Health, WHO: World Health Organization

PHQ4 Categories				
Patient characteristic	All Patients (n= 721)	Normal (n= 265, 36.8%)	Mild (n= 286, 39.8%)	Moderate to Sever (n= 170, 23.6%)	p-value	
Source of information about COVID-19, n (%)						
Healthcare authorities (MOH, WHO,...)	677 (94)	254 (95.8)	265 (92.7)	158 (92.9)	0.247	
Formal news sources & TV	390 (54.1)	141 (53.2)	153 (53.5)	96 (56.5)	0.774	
Non-formal social media (WhatsApp, Twitter, etc)	202 (28)	81 (30.6)	90 (31.5)	31 (18.2)	0.005 *	
Friends	129 (17.9)	48 (18.1)	51 (17.8)	30 (17.6)	0.992	
Not concerned or interested	45 (6.2)	8 (3)	18 (6.3	19 (11.2)	0.003 *	
Students Perception on Distance Learning, n (%)						
Did you experience distance learning (Virtual learning) before this COVID 19 pandemic?	280 (38.8)	111 (41.9)	109 (38.1)	60 (35.3)	0.368	

General knowledge in relationship to PHQ-4 categories

**Figure 1:**
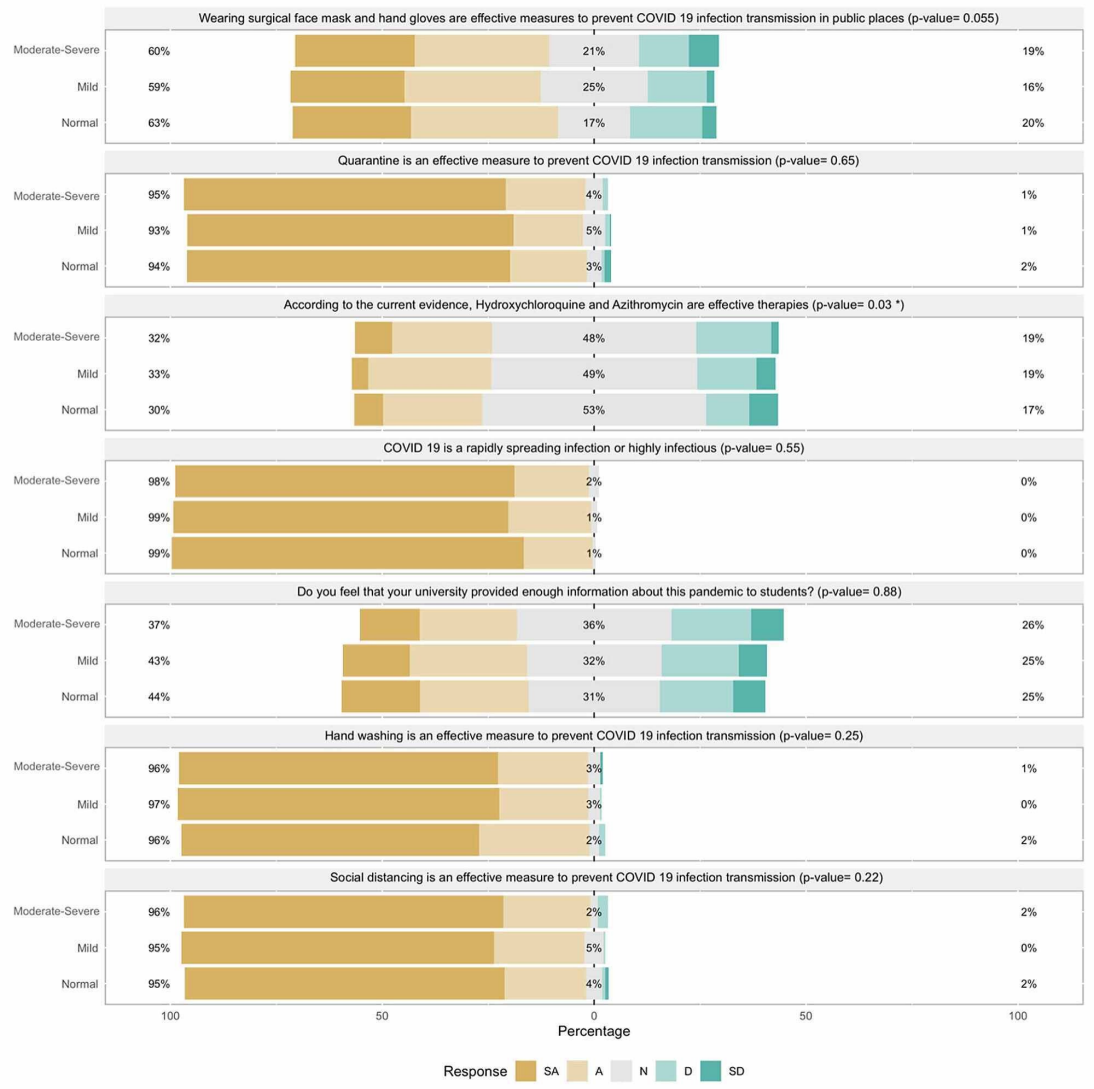
General Knowledge in relationship to Patient Health Questionnaire for Anxiety and Depression (PHQ-4) categories. *Statistically significant difference in responses between PHQ4 categories. PHQ4 Normal= 0 to 2, Mild= 3 to 5, Moderate-Severe= 6 to 12). SA: Strongly Agree, A: Agree, N: Neutral, D: Disagree, SD: Strongly disagree

When it comes to General Knowledge questions; the question “Wearing surgical face mask and hand gloves are effective measures to prevent COVID 19 infection transmission in public places” was marginally significant (p-value of trend = 0.055) with a trend of more students who scored moderate to severe in the PHQ-4 strongly disagreeing with the concept. When we asked students about the effectiveness of hydroxychloroquine and azithromycin as a treatment of COVID-19 based on current evidence; the trend was significantly different (p-value of trend = 0.03) with normal students being more likely to disagree compared to students in moderate-severe group. See Figure 1 for more details.

Students perception on distance learning in relationship to PHQ-4 categories

**Figure 2:**
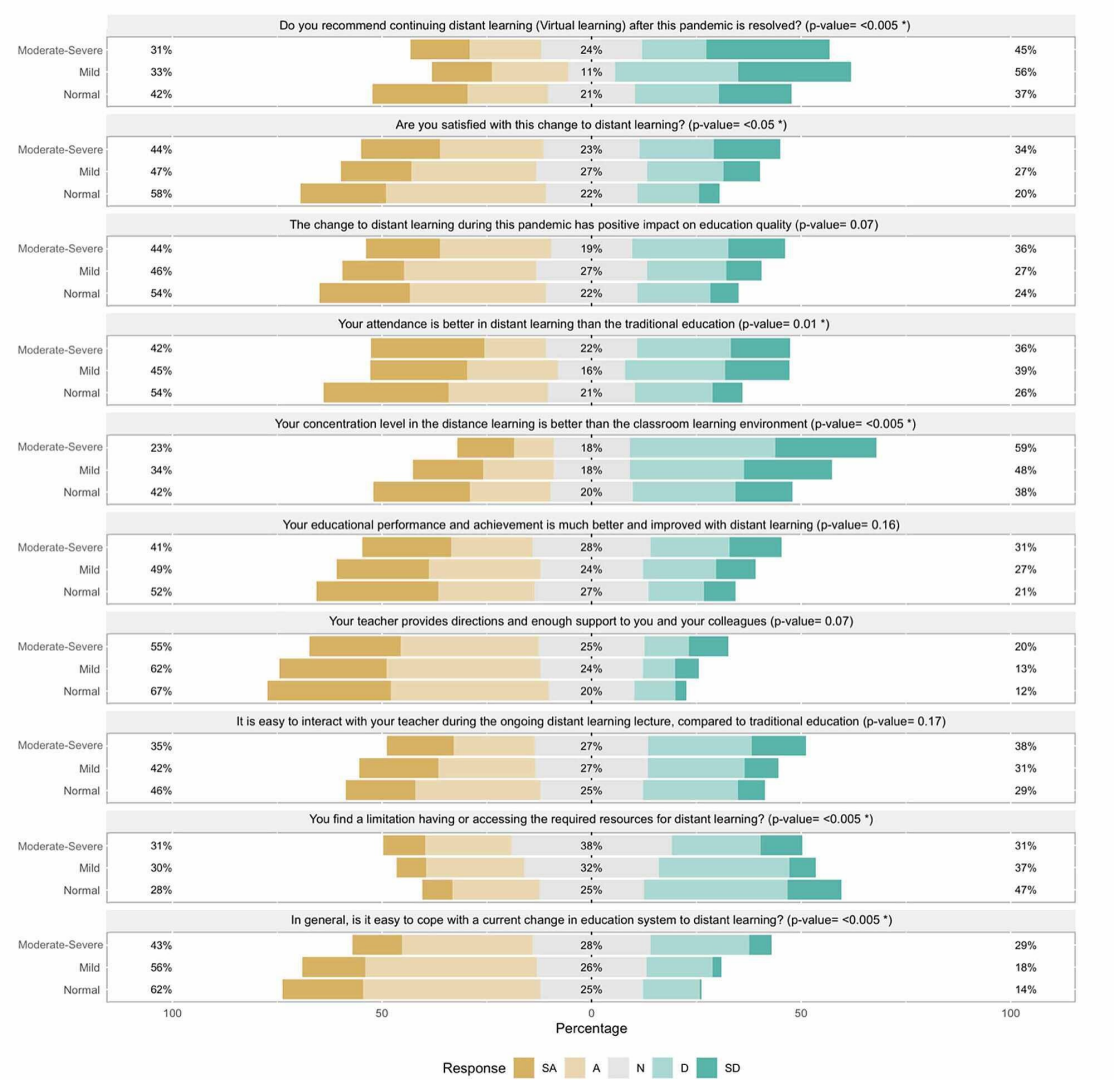
Students Perception on Distance learning in Relationship to Patient Health Questionnaire for Anxiety and Depression (PHQ-4) Categories. *Statistically significant difference in responses between PHQ4 categories. SA: Strongly Agree, A: Agree, N: Neutral, D: Disagree, SD: Strongly disagree

Normal PHQ-4 score students favor the DL approach, while the moderate to severe PHQ-4 category group dislike it. The former group expressed better concentration in DL than being in the classroom environment, while the later group disagreed and felt distracted in DL (P-value of trend = 0.003). See Figure 2 for more details.

## Discussion

Our findings showed that, during the quarantine, one-quarter of the participants had anxiety, while one-third had depression with almost 7% having a severe psychological disturbance. Age and gender were the main factors associated with higher anxiety and depression levels. Students with normal psychological assessment more often preferred DL over traditional learning methods.

The enormous leap towards DL during the COVID-19 pandemic has created contradictory opinions concerning the impact and efficacy of this method. Many explanations can be extrapolated to interpret the cause of such irreconcilable impressions. Our students’ answers were almost equally divided when asked about DL implication on their concentration, attendance, and education quality. Other studies have demonstrated that students prefer traditional over DL; however, younger generations (e.g. first-year medical students) tend to favor DL [10]. In contrast to DL, traditional or “Face-to-Face” teaching creates a more engaging environment resembling eminent concentration and meaningful discussion [11]. A possible explanation is that direct interaction gives tutors the privilege to recognize a lack of attentiveness signs where they can direct the discussion towards distracted students to recoup their focus. Furthermore, in-class teaching serves an atmosphere with lesser distraction. However, in our study, students who reported better concentration are almost similar in number compared to those who were losing concentration.

Similar findings were observed when asked about attendance and impact on the quality of teaching. Interestingly, these findings are similar to Nourian et al.'s conclusions as both traditional and DL are interchangeable in regard to knowledge acquisition [12]. However, this does not rebut previous studies where DL demonstrated better scores in exams, enhanced performance, and training efficiency [13,14]. 

Psychological conditions majorly affect the educational process. Anxiety and depression are negatively correlated with academic achievement [15]. During the COVID-19 pandemic, our study population recorded similar or slightly lower anxiety and depression rates compared to their Chinese peers [16,17]. Our findings suggest that younger age and female gender have a higher tendency to be affected by anxiety or depression. A similar observation was reported as prevalence of depression in females was 60.6% while males were 44.4% with depression being significantly higher in first-year female students [18]. Outbreaks of widespread infectious diseases by themselves are highly associated with psychological distress; nonetheless, students are at a higher risk of developing psychological disturbance [8,19]. According to our study subjects’ mean age, COVID-19 is probably the first pandemic they have encountered in adulthood. Apart from the fear of the disease itself, many regulations were adopted globally, such as social distancing, limiting gathering, travel restrictions, and curfew laws. These factors must be taken into consideration to penetrate the core of the psychological impact on the students. In Australia, during an equine influenza outbreak, individuals who were quarantined experienced higher psychological distress compared to those who were not [20], drawing attention to the effect of the isolation rather than the outbreak alone. 

To improve students’ academic progress, the psychological balance must not be relinquished. Increasing knowledge and awareness is deemed to achieve mental stability. A person’s behaviors are hugely influenced by the amount of discerned knowledge [21]. Studies on healthcare workers elaborated that anxiety level notably decreases in health professionals with better awareness [22]. The students in this study have shown adequate knowledge, especially that the vast majority has recognized that social distancing and hand washing are effective in preventing the disease. Nevertheless, a substantial number of students did not feel that their university supplied sufficient information about COVID-19. Providing an adequate amount of information about the pandemic will result in a boundless reduction in psychological agony, which eventually will improve the students’ educational accomplishments.

## Conclusions

Anxiety and depression were prevalent among healthcare students during the COVID-19 pandemic, and some students were severely affected based on the used psychological assessment tool. Also, DL is an acceptable alternative for medical education in such unavoidable pandemics as it has been noted as a preferred method for students with normal psychological assessment. Hence, we highly recommend that health science universities implement strategies and programs aiming at preventing or decreasing the psychological disorders among students as this would ensure better student wellbeing and academic performance. Further studies are required for the assessment of DL as an optimal teaching method over the traditional education method.
